# 4451 On the loss of individual joint controllability and the organization of muscle synergies in the impaired arm following a stroke: A pilot study

**DOI:** 10.1017/cts.2020.135

**Published:** 2020-07-29

**Authors:** Dongwon Kim, Kyung Koh, Raziyeh Baghi, Li-Chuan Lo, Chunyang Zhang, Dali Xu, Li-Qun Zhang

**Affiliations:** 1 University of Maryland

## Abstract

OBJECTIVES/GOALS: Damage to the sensorimotor cortex areas or/and motor/sensory pathways after a stroke could lead the motor system to a loss of controllability for joints. We investigate the loss of individual joint controllability called a loss of individualization during arm movement, which would provide an insight into abnormal motor coordination. METHODS/STUDY POPULATION: We recruit 12 chronic stroke survivors with Fugl-Meyer score between 26 and 50. A robotic exoskeleton with minimum mechanical resistance is equipped to measure the movements of the shoulder, elbow and wrist joints, respectively. Surface EMGs on muscles related to the joints are recorded using 11 wireless pre-amplified electrodes. Participants are asked to move the shoulder, elbow, or wrist joint individually throughout their range of motion, without moving the other joints voluntarily. RESULTS/ANTICIPATED RESULTS: It would be expected that participants show more difficulty in individualization of the distal joint in comparison with the proximal joint. A reduced joint range of motion would be observed in a descending order of the wrist, elbow and shoulder. These results are in line with the proximal-to-distal gradient of motor deficits after a stroke. Intention of moving the distal joint would induce a greater deviation in the position of the proximal joint than that of the distal joint when moving the proximal joint. A non-negative matrix factorization algorithm would reveal a decreased number of muscle synergies in the groups with a loss of individuation in comparison with the groups with no loss. DISCUSSION/SIGNIFICANCE OF IMPACT: We demonstrate that a stroke leads to a lack of individual joint controllability, with a greater deficits on the distal joint, and that it is related to a decreased number of muscle synergies across the corresponding joints. CONFLICT OF INTEREST DESCRIPTION: N/A.

